# Health systems strengthening interventions for perinatal common mental disorders and experiences of domestic violence in Cape Town, South Africa: protocol for a pilot implementation study

**DOI:** 10.1186/s40814-022-01053-9

**Published:** 2022-05-07

**Authors:** Zulfa Abrahams, Marguerite Schneider, Simone Honikman, Patti Olckers, Sonet Boisits, Nadine Seward, Crick Lund

**Affiliations:** 1 Alan J Flisher Centre for Public Mental Health, Department of Psychiatry and Mental Health, University of Cape Town, Building B, 46 Sawkins Road, Rondebosch, Cape Town, 7700 South Africa; 2 Perinatal Mental Health Project, Alan J Flisher Centre for Public Mental Health, Department of Psychiatry and Mental Health, University of Cape Town, Cape Town, South Africa; 3 Metro Health Services, Klipfontein/Mitchells Plain Sub Structure, Western Cape Department of Health, Cape Town, South Africa; 4 Centre for Implementation Science, Health Service and Population Research Department, Institute of Psychiatry, Psychology and Neuroscience, King’s College London, London, UK; 5 Centre for Global Mental Health, Health Service and Population Research Department, Institute of Psychiatry, Psychology and Neuroscience, King’s Global Health Institute, King’s College London, London, UK

**Keywords:** Common mental disorders, Domestic violence, Detection, Counselling, Implementation science, Health system strengthening

## Abstract

**Background:**

During the perinatal period, common mental disorders (CMDs) such as depression and anxiety are highly prevalent, especially in low-resource settings, and are associated with domestic violence, poverty, and food insecurity. Perinatal CMDs have been associated with several adverse maternal and child outcomes. While the Department of Health in South Africa provides healthcare workers with the tools to detect psychological distress and experiences of domestic violence, few healthcare workers routinely screen pregnant women at clinic visits, citing discomfort with mental health issues and the lack of standardised referral pathways as the key barriers. The aim of this study is to select and evaluate a set of health systems strengthening (HSS) interventions aimed at improving the care and outcomes for perinatal women with CMDs and experiences of domestic violence, attending public healthcare facilities in Cape Town.

**Methods:**

This study consists of a pre-implementation, development, and implementation phase. Contextual barriers identified during the pre-implementation phase included poor patient knowledge and health-seeking behaviour, high levels of stigma, and poor detection, referral, and treatment rates. Implementation science determinant frameworks were applied to findings from the pre-implementation phase to identify determinants and gaps in delivering high-quality evidence-informed care. A participatory Theory of Change workshop was used to design a HSS programme, consisting of awareness raising, detection, referral, and treatment. HSS interventions selected to support the delivery of the HSS programme includes training, health promotion, change to the healthcare environment, task-sharing, audit and feedback, and performance monitoring. The implementation phase will be used to assess several implementation and clinical outcomes associated with the delivery of the HSS programme, which will be piloted at three healthcare facilities. Qualitative and quantitative methods will be used to evaluate the implementation and clinical outcomes.

**Discussion:**

This pilot implementation study will inform us about a range of implementation and clinical outcome measures that are relevant for assessing HSS interventions for perinatal women with depression, anxiety, or experiences of domestic violence in low-resource settings. Lessons learnt from the pilot study will be incorporated into the design of a cluster randomised control trial for which further funding will be sought.

**Supplementary Information:**

The online version contains supplementary material available at 10.1186/s40814-022-01053-9.

## Background

Common mental disorders (CMDs), such as depression and anxiety, are highly prevalent during the perinatal period (pregnancy and the first year postpartum) in the Western Cape, South Africa. A facility-based study in Hanover Park, a low-income, residential suburb in Cape Town [1], reported that 21% of pregnant women were diagnosed with depression and 23% were diagnosed with anxiety [2]. Similar results were obtained from a study in Khayelitsha — a large peri-urban township settlement in Cape Town — where 19% of pregnant women screened positive for a CMD [3].

In low-resource settings such as these, the prevalence of domestic violence, poverty, and food insecurity is high. Interpersonal violence is ranked second in its contribution to the burden of disease in South Africa, and among women, intimate partner violence accounts for 62% of the burden of interpersonal violence [4]. Recent studies among perinatal women in Cape Town reported that approximately 15% of pregnant women experienced domestic violence during the past 12 months, more than 40% lived in food-insecure households, and food insecurity and domestic violence increased the risk of subsequently developing a CMD [2, 5–7]. Furthermore, perinatal CMDs have been associated with several adverse consequences for both mother and child, including preterm birth, low birthweight, and diminished mother-infant bonding [8, 9].

Following South Africa’s adoption of the Mental Health Policy Framework and Strategic Plan 2013–2020 [10], which states that mental health should be integrated into maternal and child health platforms, maternal and child health were identified by the National Department of Health (DoH) as key priority areas. With this in mind, an updated version of the Maternity Case Record (MCR) — a record book given to pregnant women and used to record all clinical details of the pregnancy [11] — containing a three-item mental health screening questionnaire [12], was released. The mental health screening questionnaire, which measures psychological distress experienced during the prior 2 weeks, was also incorporated into the Practical Approach to Care Kit (PACK) - Primary Care Guide for the Adult [13] — a clinical decision support tool for primary care workers. PACK includes guidelines for the assessment of traumatised or abused patients, and the MCR allows for healthcare workers to record the presence of abuse. However, even when provided with tools to detect psychological distress and experiences of domestic violence, few healthcare workers routinely screen pregnant women at clinic visits, citing discomfort with mental health issues and the lack of standardised referral pathways as the key barriers [14].

To assist the DoH, several models have been developed to provide screening and mental health care during the perinatal period in the Western Cape. The Perinatal Mental Health Project provides a successful screening and counselling service at one Midwife Obstetric Unit (MOU) in Cape Town [15]. The Africa Focus on Intervention Research for Mental Health (AFFIRM) randomised controlled trial developed and evaluated a structured manualised task sharing counselling intervention for antenatal depression at two MOUs in Khayelitsha, Cape Town, in 2014–2016, delivered by community health workers (CHWs) [16]. The trial showed that there was a small but significant effect of the counselling intervention on psychological distress at 3 months postnatal, compared to enhanced usual care (three supportive monthly phone calls) [3]. The effect became more significant at the 12 month postnatal assessment, suggesting that the counselling intervention has longer term benefits on managing depressive symptoms compared to the phone calls. Women in both intervention and control arms showed a substantial reduction in depression symptoms into the postnatal period. Following presentation of the preliminary AFFIRM trial results in January 2017, senior managers in the Western Cape DoH were supportive of scaling up a counselling service for antenatal depression in MOUs and basic antenatal care (BANC) clinics in the Cape Town metropolitan area.

To that end, the Health Systems Strengthening in sub-Saharan Africa (ASSET) study sought to collaborate with the Western Cape DoH to select and evaluate a set of health systems strengthening (HSS) interventions aimed at improving the quality of care and clinical outcomes for perinatal women with CMDs and experiences of domestic violence, attending public healthcare facilities in Cape Town. To help better understand how this study can be adapted for scale-up, we aim to assess the influence of the HSS interventions on important clinical and implementation outcomes such as feasibility, acceptability, fidelity, and appropriateness throughout the implementation process.

## Methods

This is a multi-phase study, consisting of a (1) pre-implementation phase, (2) development phase, and (3) implementation phase. The first two phases of the study have already been completed, and the third phase is currently in progress with enrolment in the study not yet completed.

### Implementation science

Implementation science determinant and evaluation frameworks were used throughout the study. At the end of the pre-implementation phase of the study, we used determinant frameworks to identify the barriers and/or enablers that we may have missed that would further inform the selection of any additional HSS interventions [17]. Specifically, the Theoretical Domains Framework (TDF) [18] and the Context and Implementation of Complex Interventions Framework (CICI) [19] were used to identify determinants that could potentially influence implementation efforts throughout the implementation process. The Effective Practice and Organisation of Care (EPOC) taxonomy of implementation strategies was used to select relevant HSS interventions [20]. The conceptual framework for implementation outcomes by Proctor et al. [21] was used to select relevant implementation outcomes. The TIDieR checklist and ‘Getting messier with TIDieR’ template [22] were used to describe the HSS programme in its entirety.

### Pre-implementation phase

The pre-implementation phase [23] took place in four MOUs and in the ten non-profit organisations (NPOs) mandated to provide facility- and community-based support to patients attending those facilities. The MOUs were purposively selected by DoH managers to represent each of the four sub-districts in the Cape Metropolitan health district in Cape Town. Supplementary file 1 describes the study designs, setting, data collection tools, and participants involved in the pre-implementation phase of the study. In each facility, we undertook (i) a situation analysis, (ii) a cross-sectional survey among pregnant women attending the MOUs for their first antenatal visit, (iii) qualitative interviews with pregnant women experiencing psychological distress or experiences of domestic violence, and (iv) qualitative interviews with healthcare workers. These methods and findings have been published elsewhere [14].

### Development phase

The development phase involved (i) designing a HSS programme, (ii) selecting HSS interventions to support the delivery of the HSS programme and address the contextual barriers identified during the pre-implementation phase, (iii) developing the processes and tools to deliver the HSS programme and evaluate the HSS interventions, and (iv) piloting the HSS programme at one facility.

#### Designing the HSS programme

The design of the HSS programme was guided by a Theory of Change (ToC) workshop. ToC workshops are a participatory process whereby a group of stakeholders agree on the long-term outcome the programme would like to achieve and identify the short- and medium-term outcomes needed to achieve it. The outcomes are graphically presented in a causal framework together with the assumptions of what needs to take place to achieve the outcomes, the contextual factors which influence the outcomes, and the indicators needed to measure the achievement of the outcomes [24].

**Fig. 1:**
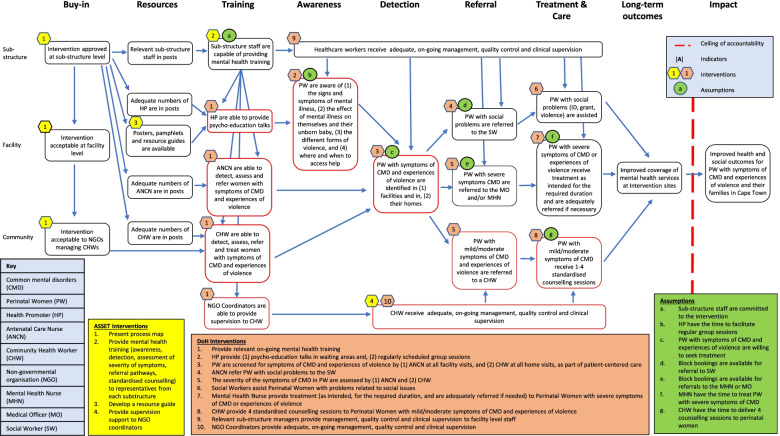
Theory of change diagram

We facilitated a ToC workshop with approximately 40 DoH managers and clinical staff working in various programmes within the four sub-districts in the Cape Metropolitan health district. Following the presentation of the pre-implementation phase findings, workshop participants discussed and agreed on the long-term outcome the programme aimed to achieve — improved coverage of maternal mental health services. Thereafter, participants worked in small groups to clarify what existed and to identify the short- and medium-term outcomes, as well as the actions and assumptions needed. Small group discussions were fed back and discussed in the bigger group. Group discussions and feedback sessions were recorded and used to guide the development of the ToC Diagram (Fig. 1). Several contextual barriers were identified including poor patient knowledge of both mental health disorders and domestic violence, high levels of stigma, poor mental health-seeking behaviour, and poor detection, referral, and treatment rates.

**Fig. 2:**
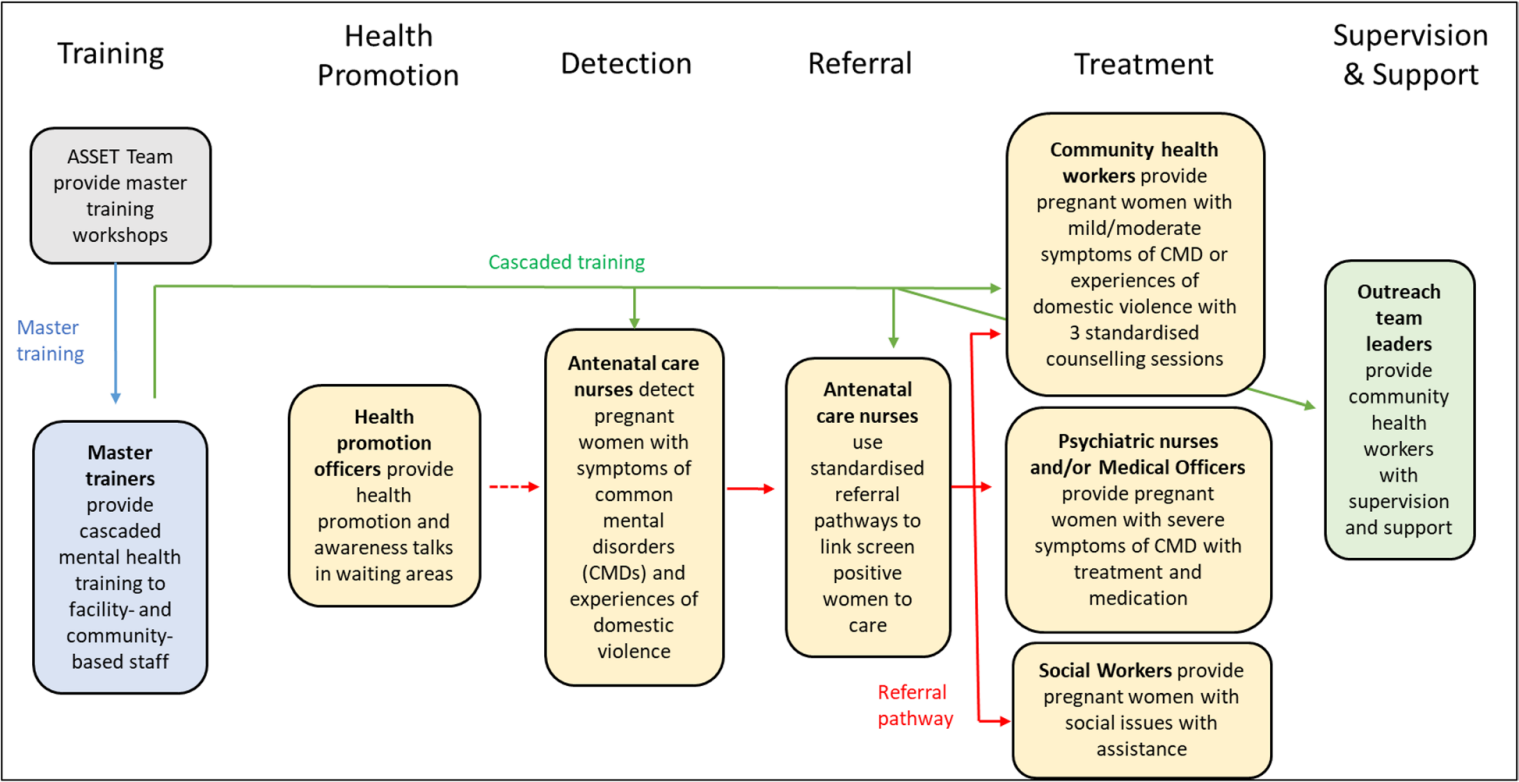
Process map for the HSS programme

The ToC diagram guided the development of a process map (Fig. 2) to visually describe the processes and actions needed by healthcare workers to achieve the long-term outcome. Over a 3-month period, an iterative process of presenting the process map to various groups of sub-district-, facility-, and community-level healthcare workers and managers for feedback, adjustment, and approval was followed.

The approved HSS programme consisted of four main components — awareness raising, detection, referral, and treatment. To operationalise the HSS programme, the following activities would be needed: (i) health promotion officers would deliver health promotion and awareness talks to pregnant women, (ii) antenatal care (ANC) nurses would detect and refer pregnant women with CMDs and experiences of domestic violence, and (iii) CHWs would deliver a structured counselling programme to pregnant women with mild to moderate symptoms of CMDs.

#### Selecting the HSS interventions and implementation outcomes

Findings from the pre-implementation phase of the study were reviewed at an annual ASSET meeting [17]. The TDF [18] and the CICI framework [19] were used to identify contextual and behavioural determinants that could influence the implementation of HSS interventions and implementation outcomes that may have been missed in the ToC workshop. If there were determinants not identified in the pre-implementation phase, these were accounted for at this stage. The HSS interventions that were selected in the ToC workshop were also labelled according to the EPOC taxonomy [18, 20]. Finally, we ensured that implementation outcomes were aligned with the selected HSS interventions. Table 1 provides an overview of the contextual barriers that were identified, the HSS interventions that were selected, and the implementation outcomes that will be assessed.

**Table 1:** Contextual barriers identified, HSS interventions selected, and implementation outcomes to be assessed during the implementation phase

Contextual barriers	HSS interventions	Implementation outcomes
Poor patient knowledge and health-seeking behaviour; high levels of stigma	**Training** — of health promotion (HP) officers to deliver health promotion talks **Health promotion** — delivery of daily health promotion talks to pregnant women	**Acceptability, appropriateness, satisfaction, and feasibility** of talks — qualitative interviews with pregnant women and HP officers **Adoption and fidelity** of talks — observation of talks and completion of a checklist **Sustainability** of daily talks — qualitative interviews with HP officers **Effectiveness** of talks — change in knowledge, attitudes, and health-seeking behaviour of pregnant women
Low levels of detection	**Training** — of antenatal care (ANC) nurses to (i) detect pregnant women with symptoms of common mental disorders (CMDs) and experiences of abuse and (ii) assess the severity of symptoms **Delivery of individual-level care** — ANC nurses screen pregnant women for symptoms of CMDs and experiences of abuse **Audit and feedback** — screening rates assessed and performance summarised and discussed at bi-monthly meetings	**Acceptability, appropriateness, satisfaction, and feasibility** of detection process — qualitative interviews with pregnant women and ANC nurses **Adoption and fidelity** of detection process — review of patient files and documents used to record detection rates **Sustainability** of the detection process — qualitative interviews with ANC nurses
Poor linkage to care	**Referral systems** — development of standardised referral pathways **Training** — of ANC nurses to refer pregnant women with symptoms of common mental disorders and experiences of abuse for treatment **Audit and feedback** — referral rates assessed and performance summarised and discussed at bi-monthly meetings	**Acceptability, appropriateness, satisfaction, and feasibility** of referral process — qualitative interviews with pregnant women and ANC nurses **Adoption and fidelity** of referral process — review of patient files and documents used for referral **Sustainability** of the referral process — qualitative interviews with ANC nurses
Limited availability of treatment	**Task-sharing** — psychological counselling programme to be delivered by lay healthcare workers **Training** — of lay healthcare workers to deliver psychological counselling **Delivery of individual-level care** — lay healthcare workers deliver psychological counselling **Change to healthcare environment** — psychological counselling delivered in patients’ homes or at off-site venues **Audit and feedback** — counselling rates assessed and performance summarised and discussed at bi-monthly meetings **Performance monitoring** — supervisors monitor delivery of counselling	**Acceptability, appropriateness, satisfaction, and feasibility** of counselling programme — qualitative interviews with pregnant women and lay healthcare workers **Fidelity** of counselling delivery — counselling competence of lay healthcare workers **Adoption, penetration, and sustainability** of counselling programme — review documents used for referral; qualitative interviews with pregnant women and lay healthcare workers

##### Developing a training model

To ensure the sustainability of the HSS programme, a cascaded training model (Supplementary file 2) was developed, whereby the ASSET team provided master training workshops at centralised locations. Three master training workshops were held with selected DoH staff members: (i) facility-based psychiatric nurses received a 1-day training on health promotion and awareness, (ii) facility PACK trainers received a 4-day training on detection and referral, and (iii) community-based services (CBS) trainers from each of the sub-districts (whose primary role was to provide on-going training to community-based healthcare workers on a range of topics) and NPO managers from the supporting NPOs received a 4-day training on counselling and a 1-day training on supervision. Master trainers were tasked with providing training to small groups of facility- and community-based healthcare workers whose role would include implementing the selected HSS interventions.

##### Addressing poor patient knowledge and health-seeking behaviour

Training and health promotion were selected as the HSS interventions to address poor patient knowledge of mental health and domestic violence, poor health-seeking behaviour, and the high levels of stigma among patients. The awareness raising component of the HSS programme will consist of health promotion officers or other lay healthcare workers providing daily, 5–7-min talks to groups of pregnant women in waiting areas at facilities. Information to be covered during the talks will include the signs, symptoms, risk factors and consequences of depression, anxiety, and experiences of domestic violence as well as the treatment options available.

Facility-based psychiatric nurses who were the recipients of the master training will be tasked with providing cascaded training to health promotion officers or other lay healthcare workers at facilities. Master trainers will be provided with a *Health Promotion and Awareness of Maternal Mental Health Training Manual* [25] to guide the delivery of the training content. Healthcare workers who will be trained to implement the health promotion talks will be provided with an A3 size, colour flipchart to guide the delivery of the talk.

##### Addressing low levels of detection

Training, delivery of individual-level care, and audit and feedback were selected as the HSS interventions to address the low levels of detection. ANC nurses will be trained to screen all pregnant women for symptoms of CMDs and experiences of domestic violence as part of routine care, using the mental health screening questionnaire [12] available in the MCR [11] and the PACK guidelines [26]. The training material was developed in conjunction with the Knowledge Translation Unit (KTU), a clinical research unit at the University of Cape Town, that led the development of PACK. The PACK guide was used as the foundation for the detection and referral process. A *PACK Antenatal Women and Mental Health Module* [27] was developed to strengthen the mental health component of routinely provided antenatal care. Four case studies formed the backbone of the module, complemented by HSS discussions, completion of relevant stationery, and a focus on effective communication strategies. PACK facility trainers were the recipients of the master training and will be tasked with training ANC nurses at their facilities. The cascaded training will consist of four, weekly, 2-hr training sessions.

During the implementation phase, audit and feedback will be used to assess the screening rates at facilities and provide feedback to the relevant ANC nurses and their managers at bi-monthly meetings.

##### Addressing poor linkage to care

Referral systems, training and audit, and feedback were selected as the HSS interventions to address the poor linkage to care. Standardised referral pathways were developed. PACK facility trainers (facility-based healthcare workers who are PACK trained and assigned to deliver PACK training to healthcare workers in their own facility) were the recipients of the master training and will be tasked with training ANC nurses at their facilities. ANC nurses will be trained to assess the severity of symptoms in pregnant women who screen positive and to use the standardised referral pathways to link them to care. Pregnant women with mild to moderate symptoms of depression will be referred to CHWs, while women with severe symptoms of depression will be referred to healthcare workers providing specialised care such as medical officers, psychiatric nurses, and psychologists. Pregnant women experiencing domestic violence will be referred to a social worker for support.

During the implementation phase, audit and feedback will be used to assess the referral rates at facilities and provide feedback to the relevant ANC nurses and their managers at bi-monthly meetings.

##### Addressing the limited availability of treatment

Several HSS interventions were selected to address the limited availability of treatment, including task-sharing, training, delivery of individual-level care, change to the healthcare environment, audit and feedback, and performance monitoring. A task-sharing psychological counselling programme was developed, to be delivered by CHWs to pregnant women with mild to moderate symptoms of depression or anxiety. It consists of three, 45-min, structured, individual-level counselling sessions using problem-solving therapy, delivered weekly by CHWs, in patients’ homes [28]. The development of the psychological counselling programme was informed by a manual review of counselling interventions, semi-structured interviews with healthcare workers and pregnant women, and finally through several stakeholder engagement meetings. CHWs will be supervised and supported by outreach team leaders (OTLs) to reinforce the counselling skills, ensure fidelity to the psychological counselling, and manage difficult cases.

CBS trainers employed in each sub-district were selected as the recipients of the master training. Master trainers received a 4-day training workshop, where they were provided with a *Counselling Skills for Community Health Workers* [29] training manual and a *Maternal Mental Health Counselling Support Guide* to assist them in delivering cascaded training on (i) counselling and (ii) supervision and support. The cascaded counselling training will be delivered over 3 days to CHWs and OTLs and consist of five sections: (i) understanding depression, anxiety, and experiences of domestic violence; (ii) basic counselling skills; (iii) patient assessment; (iv) 3-session counselling intervention; and (v) coping skills. The cascaded supervision and support training session will be delivered to OTLs during a 1-day training which will include (i) counselling support styles and skills and (ii) the use of individual and group support. CHWs and OTLs will be provided with a *Reference Guide for Community Health Workers*, in addition to the *Counselling Skills for Community Health Workers* training manual to support the delivery of the counselling intervention.

During the implementation phase, audit and feedback will be used to assess the counselling rates and provide feedback to the relevant CHWs, OTLs, and their managers at bi-monthly meetings. OTLs will also observe the delivery of the counselling sessions to monitor the performance of the CHWs.

#### Pilot study

**Fig. 3:**
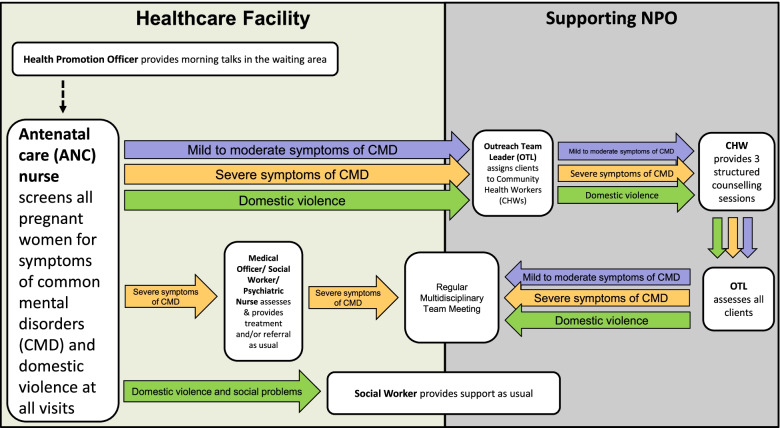
Diagram of the referral pathway

The intervention processes and tools were piloted at one of the pre-implementation phase MOUs. Lessons learnt and feedback received from healthcare workers involved in the pilot study were incorporated into the final design and used to develop a referral pathway diagram (Fig. 3).

### Implementation phase

The implementation phase will be used to deliver the HSS interventions selected to improve awareness, detection, referral, and treatment of CMDs and experiences of domestic violence in perinatal women attending public healthcare facilities between April and December 2021. Both clinical outcomes and implementation outcomes will be assessed.

#### Setting and participants

Three healthcare facilities will be purposively selected. Study sites will include a combination of (i) MOUs and BANC clinics situated in low-resource settings, (ii) provide care to women of mixed ancestry and Black African women, (iii) represent three of the four sub-districts within the Cape Metropolitan health district, and (iv) provide care to ≥60 pregnant women attending the facility for their first antenatal clinic appointment each month. All NPOs providing community-based support to the selected facilities will automatically be included.

The participants will include perinatal women attending the healthcare facilities, and sub-district-, facility-, and community-based healthcare workers. The following cadres of healthcare workers will be included: CBS trainers from each sub-district; PACK facility trainers based at each facility; psychiatric nurses, ANC nurses, and health promotion officers based at each facility; CHWs and OTLs employed by each NPOs supporting the selected facilities.

#### Study designs

The following study designs will be used to assess the implementation and clinical outcomes of the intervention: (i) healthcare worker survey, (ii) patient survey, (iii) observation of health promotion talks, (iv) cohort study, (v) patient file reviews, (vi) documentation review, (vii) counselling competence, and (viii) qualitative assessments. One fieldworker will be based at each of the three facilities and be responsible for all the data collection. Anticipated sample sizes are based on experience gained during the pre-implementation phase, as well as on the goal of assessing the feasibility and acceptability of the various HSS intervention components and related measures. Table 2 provides an overview of the study designs and objectives, data collection instruments, participants to be recruited, and the timing of data collection.

**Table 2:** Implementation phase study designs, study objectives, data collection tools, study participants, and timing of data collection

Study design	Study objectives	Data collection tools	Participants	Timing of data collection
Healthcare worker survey	Assess changes in healthcare workers’ knowledge of mental illness and domestic violence and their attitudes towards people with mental health disorders and experiences of domestic violence	Self-administered questionnaires: organisational readiness for implementing change [30]; bespoke knowledge questionnaire; mental illness: Clinicians’ Attitudes Scale [31]; Professional Quality of Life Scale [32]; Effort-Reward Imbalance scale [33]	All healthcare workers involved in the intervention — CBS trainers, NPO managers, PACK facility trainers, psychiatric nurses, ANC nurses, OTLs, CHWs, health promotion officers, HIV counsellors, nursing assistants	Two timepoints — before receiving training and at the end of the implementation phase
Patient survey	Assess changes in pregnant women’s knowledge of mental illness and domestic violence and their attitudes towards people with mental health disorders and experiences of domestic violence	Self-administered bespoke questionnaire consisting of 16 questions	Pregnant women attending MOUs and BANC clinics	Two timepoints — before the delivery of health promotion talks and after delivery of the health promotion talks
Observation of health promotion talks	Assess uptake, fidelity, penetration, and sustainability of health promotion talks	Checklist to be completed by fieldworkers observing the talks		During the delivery of health promotion talks
Cohort study	Assess the presence of and risk factors for CMDs and experiences of domestic violence; assess whether the HSS interventions to improve awareness, detection, referral, and treatment resulted in improvement in clinical outcomes	Interviewer-administered questionnaires: Edinburgh postnatal depression scale [34]; psychological distress screening tool [12]; bespoke questionnaire to assess domestic violence; Household Food Insecurity and Access Scale [35]; Composite Abuse Scale [36]; WHO Disability Assessment Schedule [37]; Multidimensional Scale of Perceived Social Support [38]; Mother-to-Infant Bonding Scale [39]	Pregnant and postnatal women attending antenatal care clinics	Three timepoints — (1) when pregnant women are recruited, (2) when participants are 36 weeks pregnant, and (3) 6 weeks after participants have given birth
Patient file reviews	Assess changes in detection and referral rates of pregnant women with CMDs and experiences of domestic violence	Maternity case record [11]	Pregnant and postnatal women attending MOUs and BANC clinics	Weekly during the implementation phase
Documentation review	Assess coverage of the detection, referral, and treatment interventions	Bespoke patient registers and tracking forms	ANC nurses and OTLs	Daily during the intervention period
Counselling competence	Assess fidelity to the structured counselling format and content	Enhancing assessment of common therapeutic factors tool [40]; bespoke counselling evaluation form	Community health workers (CHWs)	During the last 2 months of the intervention
Qualitative assessments	Assess the acceptability, appropriateness, satisfaction, and feasibility of the selected HSS interventions	Topic guides for key informant interviews and focus group discussions	Pregnant and postnatal women involved in the intervention Healthcare workers involved in the intervention	During the last 3 months of the intervention

##### Healthcare worker survey

All healthcare workers involved in the implementation of a HSS intervention will be asked to complete a self-administered survey questionnaire prior to receiving training. The questionnaire will be available in participants’ home language and take 30 to 45 min to complete. In the last month of the study, healthcare workers who were actively involved in the intervention will be asked to complete the same survey questionnaire. We expect the number of healthcare workers linked to each facility to differ but estimate that between 10 and 20 healthcare workers are linked to each of the MOUs and BANC clinics. We intend to approach all healthcare workers and anticipate that at least 70% (i.e. 7–10 healthcare workers per facility) will agree to participate in the survey. The survey will be used to evaluate whether delivering the various HSS interventions resulted in a change in healthcare workers’ mental health knowledge, attitudes towards persons with mental illness, their psycho-social well-being, and their quality of life.

##### Patient survey

During the baseline data collection period (prior to delivering the HSS interventions), pregnant women awaiting their routine tests and consultations will be approached and asked to complete a self-administered survey questionnaire, available in their home language. During the implementation phase, pregnant women who received the health promotion talk will be asked to complete the same survey questionnaire immediately after the talk has been delivered. The survey questionnaire will consist of a few demographic questions; questions about their knowledge of depression, anxiety, and domestic violence; questions on attitudes towards people with mental illness and experiences of domestic violence; and questions on their health-seeking behaviour. We anticipate that 15 to 30 pregnant women will be willing to complete the survey after a talk and plan on administering the survey daily during the 2 weeks of baseline data collection and at least once per week during the implementation phase. The survey will be used to assess the effect of the health promotion talks in changing the mental health knowledge, attitudes, and health-seeking behaviour of perinatal women.

##### Observation of health promotion talks

Fieldworkers based at facilities will be tasked with observing the delivery of the health promotion talks and completing a checklist to assess (i) the extent to which the talks are adopted at facilities, (ii) whether the talks are being delivered as intended, and (iii) the extent to which the talks are accessed by the pregnant women.

##### Cohort study

At baseline, pregnant women attending the clinic for their first antenatal visit will be screened by fieldworkers, in their home language, for the presence of CMDs and experiences of domestic violence. All women who screen positive — i.e. score ≥13 on the EPDS or ≥2 on the bespoke violence screening tool — will be invited to participate in the cohort study. A random sample of 33% of pregnant women who screen negative (identified by Redcap, using a randomisation table) will also be invited to participate in the cohort study. Fieldworkers will administer several questionnaires assessing risk factors for CMDs and the frequency of domestic violence. The presence of and risk factors for CMDs and experiences of domestic violence in cohort participants will be assessed again at the 36-week gestation and the 6-week postnatal follow-up timepoints. Changes in clinical outcomes and their risk factors will be assessed by comparing the 36-week gestation and the 6-week postnatal outcomes to the baseline outcomes.

The cohort study will be used (i) to assess the presence of and risk factors for CMDs and experiences of domestic violence in pregnant women and (ii) to assess whether the HSS interventions resulted in improvement in clinical outcomes. It will be based on the proportion of women showing a clinically significant improvement as measured by the EPDS. Women with an EPDS score of ≥13 at baseline will be classified as having symptoms of CMD. Clinically significant improvement will be classified as having a 50% reduction in EPDS scores measured at the 36-week gestation and 6-week postnatal follow-up timepoints, compared to baseline. The study will also assess the feasibility of administering these instruments and effectively following up perinatal women in these communities, for a potential future cRCT.

Sample size calculations were used to determine the number of participants needed for a cluster randomised control trial (cRCT). Based on a level of significance (alpha) = 0.05, an intra-cluster correlation coefficient (ICC) = 0.02, an effect size = 0.27, the correlation coefficient between baseline and follow-up (*r*) = 0.3–0.5, and power = 80–90%, 225–291 participants would be needed at each of the intervention and control facilities in a cRCT. For this pilot study at three facilities, we intend to recruit 225–291 participants per facility. If we are able to recruit 20 perinatal women per week at each facility, we would need to spend at least 3 months doing recruitment.

##### Patient file reviews

During the baseline data collection period and throughout the implementation phase, fieldworkers will capture the outcome of the psychological distress questionnaire found in the MCRs of pregnant women attending healthcare facilities. The information obtained will be used to assess whether delivering the HSS interventions improved (i) detection and referral rates, (ii) uptake of the detection and referral interventions, and (iii) the sustainability of the detection and referral interventions.

##### Documentation reviews

Reviewing registers and referral forms will be used to assess the coverage of the detection, referral, and treatment interventions. Rates of detection and referral for women with CMDs and experiences of domestic violence will be assessed before the intervention, and monitored weekly throughout the intervention period, by triangulating information obtained from (i) a review of the daily patient registers completed by ANC nurses and (ii) reviewing the referral forms completed by ANC nurses for patients who screen positive and agree to counselling.

The proportion of perinatal women with CMDs and experiences of domestic violence who take up the referral and receive partial or full treatment will be assessed by triangulating information obtained from reviewing the referral and counselling feedback forms to be completed by OTLs for all patients who are referred for the task-sharing psychological counselling programme.

##### Counselling competence

The counselling competence of the CHWs delivering the counselling intervention will be measured during their first three counselling sessions. OTLs will be tasked with completing a counselling session evaluation form while observing the counselling sessions. The evaluation form will use a Likert scale and consist of questions assessing their verbal and non-verbal skills, as well as a checklist to evaluate the fidelity to the structured counselling format and content.

##### Qualitative assessments

Key informant interviews and focus group discussions with perinatal women, ANC nurses, CHWs, and OTLs will be used to assess the acceptability, appropriateness, satisfaction, and feasibility of the various HSS interventions.

#### Ethical approval and consent to participate

Ethical approval for the study has been obtained from the Human Research Ethics Committee at the University of Cape Town as well as from the Psychiatry, Nursing and Midwifery Research Ethics Subcommittee at King’s College London. In addition, the Western Cape Department of Health approved the use of the research sites. All participants will be asked to provide written, informed consent after the procedure is verbally explained to them. Participants will be informed of their right to withdraw from the study at any time without consequences. Pregnant women who are recruited into the cohort study will receive a ZAR100 food voucher after completing all questionnaires at recruitment and again at the 36-week follow-up interview.

#### Data handling and confidentiality

All data will be processed in accordance with the General Data Protection Regulation 2018 (GDPR). Participants will be allocated a unique identifier, which will be used to maintain confidentiality and minimise the use of personal information. Questionnaires completed in hard copy will be stored in a locked room at the University of Cape Town. King’s College London will be the data controller for the ASSET study.

#### Data analyses

A combination of mixed methods will be used to analyse the data collected. REDCap, a secure web platform for building and managing online databases and surveys, will be used to capture all quantitative data. Data will be exported to STATA/SE statistical software package version 15.1 (StataCorp., College Station, TX, USA) for analysis. Multiple imputation will be used to account for participants with incomplete data. Categorical variables will be described using frequency and percentages, and associations measured using chi-square tests. Continuous variables will be described using means and standard deviations, and associations measured using *t*-tests. Linear regression models will be used to evaluate for change in EPDS scores, adjusting for the clinic and any potential confounders. Implementation outcomes will be evaluated using multivariable regression models that adjust for relevant confounders and clinic.

Qualitative data will be analysed using NVivo 12 Pro qualitative data analysis software (QSR International Pty Ltd) [41]. Semi-structured interviews will be transcribed by bilingual speakers. Transcripts will be analysed using a thematic analysis approach to generate initial codes and define, search for, and review themes [42]. The development of initial codes will be guided to a certain degree by the interview topic guides. Further themes not captured by the initial coding will be identified through extensive reading of the transcripts and coding passages interpreted as important.

## Discussion

There is a substantial treatment gap for perinatal women with depression, anxiety, and exposure to domestic violence in low-resource African settings. We selected HSS interventions to improve awareness, detection, referral, and treatment of perinatal women experiencing symptoms of CMDs and domestic violence in Cape Town, South Africa. The purpose of this protocol is to describe how we will assess the effect of the HSS interventions on important implementation outcomes. The findings will help us refine the intervention components by identifying the barriers and facilitators experienced by the healthcare providers, their managers, and the patients receiving the intervention. While we aim to improve patient knowledge of domestic violence, and thereby improve their health-seeking behaviour, we do not intend to manage active cases of domestic violence by engaging with the perpetrators.

The findings will also inform us about the feasibility and acceptability of a variety of implementation and clinical outcome measures in this context. Lessons learnt from the pilot study will be incorporated into the design of a cRCT for which further funding will be sought. The integration of detection, referral, and care for perinatal women with depression, anxiety, and exposure to domestic violence into routine low-resource health facility and community settings is complex and requires a careful evaluation of process and outcomes, if it is to be scaled up for broader population benefit.

## Supplementary Information

**Additional file 1:** Overview of study designs used during the Pre-Implementation Phase [43].

**Additional file 2:** Overview of the cascaded training model.

## Data Availability

The datasets used and/or analysed during the current study are available from the corresponding author on reasonable request.

## Abbreviations

AFFIRM: Africa Focus on Intervention Research for Mental Health
ANC: Antenatal care
BANC: Basic antenatal care
CBS: Community-based services
CHW: Community health worker
CMD: Common mental disorder
DoH: Department of Health
EPDS: Edinburgh Postnatal Depression Scale
EPOC: Effective Practice and Organisation of Care
HSS: Health systems strengthening
KTU: Knowledge Translation Unit
MOU: Midwife Obstetric Unit
MCR: Maternity case record
NPO: Not-for-profit organisation
OTL: Outreach team leader
PACK: Practical approach to care kit
TDF: Theoretical Domains Framework
ToC: Theory of change
WC: Western Cape
