# Supplementary material for: Health systems strengthening interventions for perinatal common mental disorders and experiences of domestic violence in Cape Town, South Africa: protocol for a pilot implementation study
Source: Pilot Feasibility Stud. 2022 May 7;8:100. doi: 10.1186/s40814-022-01053-9 (PMC9077881; doi:10.1186/s40814-022-01053-9)
Supplement: Supplementary file 1 — Additional file 1: Overview of study designs used during the Pre-Implementation Phase [43]. [file 40814_2022_1053_MOESM1_ESM.docx]

**Supplementary file 1**: Overview of study designs used during the Pre-Implementation Phase

| **Study design** | **Objectives** | **Data collection tools** | **Setting** | **Participants** |
| --- | --- | --- | --- | --- |
| Situation Analysis | Assess governance, organisation and leadership of care processes; healthcare worker numbers, roles, skills and competencies; availability and use of policies and guidelines; patient flow through the health system; availability and functionality of essential equipment; availability of essential drugs; functioning of the health system information management system, and to assess routinely collected data | Adapted version of the situation analysis tool developed by the Programme for Improving Mental Health Care (PRIME) [43] | Four purposively selected midwife obstetric units (MOUs) in the Cape Metropolitan health district and 10 not-for profit organisations (NPOs) providing community- and facility-based support to the MOUs | 12 facility- and community-based managers employed by the Department of Health (DoH) or supporting NPOs |
| Qualitative healthcare worker interviews | Assess healthcare worker roles and attitudes to detecting, referring and treating pregnant women with CMDs and experiences of domestic violence; thoughts on the acceptability and feasibility of providing a routine screening and counselling service for pregnant women | Semi-structured interview guide | Four purposively selected midwife obstetric units (MOUs) and 10 not-for profit organisations (NPOs) providing community- and facility-based support to them in the Cape Metropolitan health district | 37 facility- and community-based healthcare workers employed by the Department of Health (DoH) or supporting NPOs |
| Quantitative patient survey | Assess the prevalence of CMDs and experiences of domestic violence in pregnant women | Bespoke questionnaire to collect socio-demographic information  Edinburgh postnatal depression scale [25]  Validated psychological distress screening tool [12]  Bespoke screening tool to collect information on experiences of domestic violence | Four purposively selected midwife obstetric units (MOUs) in the Cape Metropolitan health district | 156 pregnant women attending the MOUs for their first antenatal visit |
| Qualitative patient interviews | Assess experiences of depression, anxiety and domestic violence; perceived reasons for experiencing psychological distress; thoughts on the acceptability and feasibility of a routine screening and counselling service for pregnant women | Semi-structured interview guide | Four purposively selected midwife obstetric units (MOUs) in the Cape Metropolitan health district | 38 pregnant women attending the MOU for their first antenatal visit |
