# Supplementary figures and images for: Health systems strengthening interventions for perinatal common mental disorders and experiences of domestic violence in Cape Town, South Africa: protocol for a pilot implementation study
Source: Pilot Feasibility Stud. 2022 May 7;8:100. doi: 10.1186/s40814-022-01053-9 (PMC9077881; doi:10.1186/s40814-022-01053-9)

**Supplementary file 2**: Cascaded training model


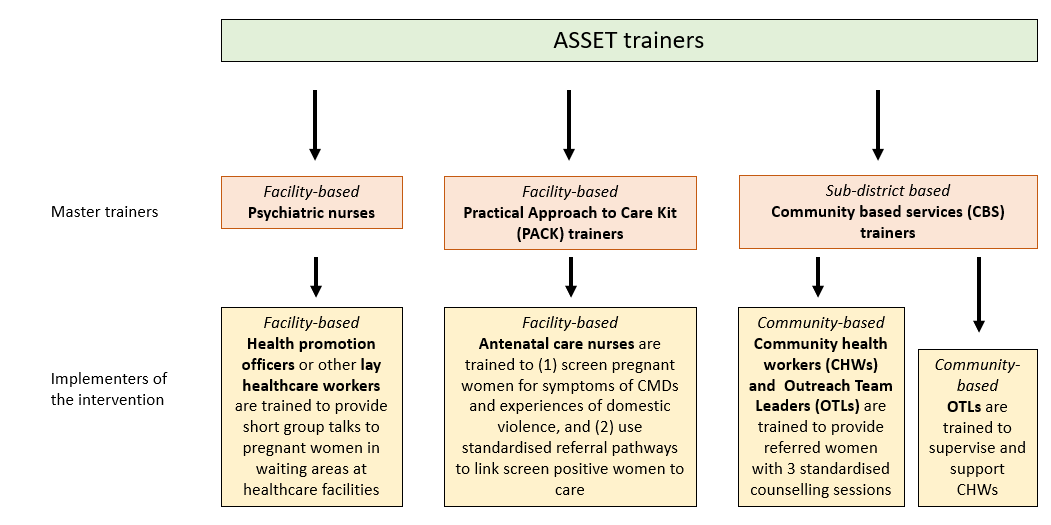

Supplement: Supplementary file 2 — Additional file 2: Overview of the cascaded training model. [file 40814_2022_1053_MOESM2_ESM.docx]
